# Metabolic Engineering of Oleaginous Yeasts for Production of Fuels and Chemicals

**DOI:** 10.3389/fmicb.2017.02185

**Published:** 2017-11-08

**Authors:** Shuobo Shi, Huimin Zhao

**Affiliations:** ^1^Beijing Advanced Innovation Center for Soft Matter Science and Engineering, Beijing University of Chemical Technology, Beijing, China; ^2^Metabolic Engineering Research Laboratory, Science and Engineering Institutes, Agency for Science, Technology and Research, Singapore, Singapore; ^3^Department of Chemical and Biomolecular Engineering, University of Illinois at Urbana-Champaign, Urbana, IL, United States

**Keywords:** metabolic engineering, oleaginous yeasts, fuels, chemicals, fatty acids

## Abstract

Oleaginous yeasts have been increasingly explored for production of chemicals and fuels via metabolic engineering. Particularly, there is a growing interest in using oleaginous yeasts for the synthesis of lipid-related products due to their high lipogenesis capability, robustness, and ability to utilize a variety of substrates. Most of the metabolic engineering studies in oleaginous yeasts focused on *Yarrowia* that already has plenty of genetic engineering tools. However, recent advances in systems biology and synthetic biology have provided new strategies and tools to engineer those oleaginous yeasts that have naturally high lipid accumulation but lack genetic tools, such as *Rhodosporidium*, *Trichosporon*, and *Lipomyces*. This review highlights recent accomplishments in metabolic engineering of oleaginous yeasts and recent advances in the development of genetic engineering tools in oleaginous yeasts within the last 3 years.

## Introduction

A wide range of molecules can be produced by microorganisms, including amino acids, organic acids, polymers, alcohols, ethers, esters, isoprenes, alkenes, and alkanes (Du et al., 2011; Liu L. et al., 2013; Cordova and Alper, 2016; Liao et al., 2016; Chen et al., 2017). It is worthy to note that the versatility of fatty acids (FAs) has led to the synthesis of a wide variety of industrially important compounds. These range from relatively low-volume, high-value products [e.g., polyunsaturated FAs, PUFAs] to high-volume, low value products such as biofuels or oleochemicals. FA-derived chemicals, including free FAs (FFAs) (Zhou et al., 2016), fatty alcohols (FALs) (Feng et al., 2015), FA ethyl esters (FAEEs) (Steen et al., 2010; Shi et al., 2012), or FA methyl esters (FAMEs) (Nawabi et al., 2011), and fatty alkane/alkenes (Schirmer et al., 2010), are of particular interest, since they fulfill a role as platform molecules of a cluster of important fuels. The global market for natural FAs was predicted to reach $16.2 billion by 2021 from $12.4 billion in 2016 at a compound annual growth rate (CAGR) of 5.6% (McWilliams, 2017). The derivatives market may reach $8.5 billion by 2021 from $6.2 billion in 2016 at a CAGR of 6.8% (McWilliams, 2017).

There is a significant amount of studies focusing on increasing the metabolic flux through the FA biosynthetic pathway in model microorganisms such as *Escherichia coli* (Schirmer et al., 2010; Steen et al., 2010; Xu et al., 2014) and *Saccharomyces cerevisiae* (Runguphan and Keasling, 2013; Lian and Zhao, 2015; Zhou et al., 2016). However, enhancing FA biosynthesis is usually difficult due to tight and complex regulation and essential roles of FA in normal physiology, and limited improvements were obtained in previous studies. Instead, an increasingly number of studies have explored oleaginous microorganisms for production of FA-derived compounds (Levering et al., 2015; Qadeer et al., 2017), considering that they have naturally evolved to harbor high levels of triacylglycerols (TAGs) and FFAs (more than 30% lipid in dry cell weight). Of these, oleaginous yeasts have attracted considerable interest for production of FA-related products (Probst et al., 2016; Adrio, 2017). Oleaginous yeasts are advantageous because they can quickly grow to high densities with a high lipid content and their cultures are more easily scaled up in an arable land-independent and controllable manner. Furthermore, their ability to utilize a large number of renewable substrates and inexpensive materials make oleaginous yeasts economically interesting. Besides, their ability to have good growth at low pH is a considerable advantage in preventing bacterial contamination, which facilitate the process development for future industrial applications. Since the supply of acetyl-CoA is efficient in oleaginous yeasts, it is also proposed to serve as cell factories to produce other acetyl-CoA derivative products, e.g., poly-3-hydroxybutyrate (PHB) (Li Z.J. et al., 2016) and terpenoids (Vickers et al., 2017).

Oleaginous yeast strains identified so far mainly include species belonging to the genera *Yarrowia*, *Candida*, *Rhodotorula*, *Rhodosporidium*, *Cryptococcus*, *Trichosporon*, and *Lipomyces* (Ageitos et al., 2011). Screenings for novel oleaginous yeasts are still being performed, leading to the identification of novel oleaginous strains (Lamers et al., 2016; Viñarta et al., 2016). Among oleaginous yeasts, *Yarrowia lipolytica* is the most-well studied one. Thanks to the availability of genetic tools (Madzak, 2015; Schwartz et al., 2015), *Y. lipolytica* has been used for a variety of biotechnological applications, including the production of PUFAs (Xue et al., 2013; Liu et al., 2017; Sun et al., 2017), citric acid (Förster et al., 2007; Moeller et al., 2013; Tan et al., 2016), and alkanes (Xu et al., 2016). Moreover, *Y. lipolytica* has also been shown to be rather robust and able to grow on a variety of substrates (Papanikolaou et al., 2002; Ledesma-Amaro and Nicaud, 2016b; Mirończuk et al., 2016). For example, despite the high contamination, crude glycerol is easily utilized by the yeast *Y. lipolytica* (Papanikolaou et al., 2002). Recently, extensive efforts have been made to understand the genetics and physiology of *Y. lipolytica* (Pomraning et al., 2015; Kerkhoven et al., 2016; Ledesma-Amaro and Nicaud, 2016a; Lazar et al., 2017), allowing accelerated metabolic engineering efforts for a variety of different products.

Compared with *Y. lipolytica*, the metabolic engineering of other oleaginous yeasts is still limited due to the lack of genetic tools and generally insufficient knowledge of cellular genetics. Currently, *Rhodosporidium*, *Trichosporon*, and *Lipomyces* have been considered as attractive hosts because these genera have shown higher lipid accumulation (over 60%) and adaptability to consume a wide range of substrates in the feedstock utilization (Li et al., 2007; Lin et al., 2011; Freitas et al., 2014; Kourist et al., 2015; Görner et al., 2016). It is also of importance to note that the genus *Rhodosporidium* is a good producer of carotenoids (Buzzini et al., 2007), and shows excellent tolerance toward inhibitory compounds found in biomass hydrolysates (Hu et al., 2009). The recent establishment of their genetic accessibilities paved the way for developing a more economically feasible lipid production process in these yeasts (Görner et al., 2016; Liu et al., 2016; Tsai et al., 2016; Lin et al., 2017). However, more advanced genetic tools and better understanding of the genetics are needed if the full potential of these non-conventional yeasts as platforms for producing lipid-based chemicals is to be realized.

In this review, we will summarize and interpret the current trends in engineering oleaginous yeasts for the production of fuels and chemicals within the last 3 years. A significant difference of this review compared to other related recent reviews (Probst et al., 2016; Adrio, 2017) is that this review focuses on the recent advances in not only the production of FA-related products, but also the production of non-FA and some unique products and engineering strategies for the utilization of various substrates. Particularly, we will highlight the recent advances in developing facile and efficient genetic tools in oleaginous yeasts. Finally, we will provide a prospective on the industrial application of these promising oleaginous yeasts.

## Development of Tools and Approaches for Production of Fuels and Chemicals in Oleaginous Yeasts

Many basic and advanced genetic tools have been developed in oleaginous yeasts, especially in *Y. lipolytica.* However, the existing tools are still limited in oleaginous yeasts to allow efficient genetic engineering. To date, a wide variety of new strategies and tools are under development.

### Genetic Elements

Basic genetic elements, including development of different promoters, vectors, selection markers, and so on, have been developed in oleaginous yeasts to facilitate metabolic engineering application. *Y. lipolytica* is the oleaginous yeast with the most available genetic elements. These genetic elements in *Y. lipolytica* include an efficient one-step transformation method (Chen et al., 1997); promoters for constitutively expressing genes at varied levels (Madzak et al., 2000; Blazeck et al., 2011; Tai and Stephanopoulos, 2013) or to be induced/repressed under certain conditions (Gasmi et al., 2011; Braun et al., 2012); vectors with different copy numbers (Liu et al., 2014), as well as auxotrophic and antibiotic resistance markers (Fickers et al., 2003; Blazeck et al., 2011). Generally, promoter elements are among the first to be annotated and developed for new hosts. Shabbir Hussain et al. (2015) systematically examined various promoter components including upstream activating sequences (UAS), proximal promoter sequences, core promoters, and the TATA box, and found that the strength of promoter could be controlled by engineering the TATA box sequence, core promoter, and UAS. Similarly, promoters were rationally created with various numbers of UAS1 tandem elements, and it was reported that the gene expression increased as the number of UAS1 tandem elements increased (Dulermo et al., 2017), similar to what was reported in *S. cerevisiae* (Blazeck et al., 2012). Recently, a panel of terminators (ranging from 35 to 70 bp) that can regulate gene expression were also developed in *Y. lipolytica* (Curran et al., 2015). The best of these synthetic terminator resulted in a 3.7-fold more fluorescent protein output and a 4.4-fold increase in transcript level compared to the commonly used 240 bp *CYC1* terminator, representing a movement toward short, minimal synthetic part. All these findings enabled the design of a fine-tuning system for gene expression in *Y. lipolytica*.

There is a growing interest in using other oleaginous yeasts, such as *Rhodosporidium toruloides*, *Lipomyces starkeyi*, and *Trichosporon oleaginosus*, due to their much higher lipid content (over 60% of biomass). However, their rational genetic engineering is impeded by the lack of efficient genetic manipulation methods and genetic elements. Therefore, efforts are required to establish efficient genetic elements in these yeasts.

The first transformation method developed for *R. toruloides* was the spheroplast–polyethylene glycol (PEG) transformation method (Tully and Gilbert, 1985). However, the method was limited by the low efficiency, unstable chromosomal integration, and auxotrophic selection. An *Agrobacterium*-mediated transformation (AMT) method was developed to improve the transformation efficiency in *R. toruloides* (Liu Y. et al., 2013). The AMT method was used to integrate multiple genes into the chromosome simultaneously in *R. toruloides* (Lin et al., 2014). These methods relied on the use of several genetic elements, such as the strong promoters (e.g., GPDp and PGKp) (Liu Y. et al., 2013; Lin et al., 2014), plasmid vectors (Liu Y. et al., 2013; Lin et al., 2014), and antibiotic resistance markers (e.g., hygromycin, nourseothricin, and bleomycin) (Lin et al., 2014). In *R. toruloides*, it was found that the recently isolated *DAO1* promoter could be strongly induced when D-amino acids were provided (Liu et al., 2015d). However, the basal expression level under non-inducing conditions remained high. Additionally, four inducible promoters, NAR1p, ICL1p, CTR3p, and MET16p, were identified by screening with promoter-EGFP reporters in *R. toruloides* (Johns et al., 2016). Each promoter had its own individual characteristics for controllable gene expression in particular applications. Later, the promoters of six genes involved in lipid biosynthesis or accumulation were analyzed (Liu et al., 2016). Among them, the *LDP1* promoter displayed much stronger activity (4- to 11-folds) than that of the glyceraldehyde-3-phosphate dehydrogenase gene (*GPD1*), one of the strongest promoters known in yeasts. The *LDP1* promoter was successfully used to drive *DGA1* gene for enhanced lipid accumulation. In a related study, five different constitutive promoters were cloned and evaluated in *R. toruloides* (Wang et al., 2016c). The strength of these promoters was demonstrated at both the phenotypic level and the transcriptional level, and it was reported that the promoter strength followed a decreasing order from *PGIp*, *PGKp*, *FBAp*, *TPIp*, to *GPDp*.

Recently, several transformation methods were developed for *L. starkeyi*, such as a lithium acetate transformation method (Calvey et al., 2014), a PEG transformation method (Oguro et al., 2015), and an AMT method (Lin et al., 2017). The latest AMT method was simpler and more convenient with fewer steps. In addition, in this study, two exogenous constitutive promoters, the GPD promoter, and the PGK promoter, were demonstrated to be functional, enabling *L. starkeyi* to serve as a unique oleaginous yeast for further metabolic engineering studies.

The first transformation protocol for *T. oleaginosus* also used the AMT method (Görner et al., 2016). Strong expression of a heterologous YFP reporter was achieved by using the constitutive promoter from the endogenous GPD gene. The genetic elements are still lacking in these yeasts, and a variety of genetic tools should be developed to facilitate strain development activities.

### Rapid Assembly and Integration of Metabolic Pathways

In synthetic biology, different combinations of genetic elements are used to create metabolic pathways with desired properties. Traditional methods employ the classic restriction digestion and ligation method for pathway construction, which is time-consuming and expensive. Currently, various DNA assembly and integration methods are available for constructing pathways, such as DNA assembler (Shao et al., 2009), ePathBrick (Xu et al., 2012), LCR assembly (De et al., 2014), Golden Gate assembly (Agmon et al., 2015), CasSEMBLR (Jakočiūnas et al., 2015), CrEdit (Ronda et al., 2015), and Di-CRISPR (Shi et al., 2016). Generally, these methods are mainly applied to *E. coli* and *S. cerevisiae*, and are rarely applied to non-conventional yeasts.

As a proof of concept, an entire β-carotene biosynthesis pathway with multiple fragments (four genes with a total size of ∼11 kb) were assembled via *in vivo* homologous recombination (HR) into rDNA locus, which is a tandem repeat identified in *Y. lipolytica* (Gao et al., 2014). Similarly, using rDNA as integrative sites, the biosynthetic pathway of arachidonic acid (ARA) was assembled and integrated to *Y. lipolytica* in one-step (Liu et al., 2017). The resulting pathway showed long-term genetic stability and enabled the strain to produce ARA at 0.4% of total lipid. In another work, Golden Gate assembly was established in *Y. lipolytica* (Celińska et al., 2017). A broad set of destination vectors and interchangeable building blocks were constructed as Golden Gate bricks. This technology was used to construct the synthetic pathway for carotenoid production, and the efficiency was significantly improved (up to 90%). This high efficiency can reduce the time and workload, permit faster and accurate multiple target engineering, as well as guarantee standardization for modular DNA cloning techniques. Later, a CRISPR–Cas9-based tool was developed in *Y. lipolytica* for targeted, markerless gene integration (Schwartz et al., 2017). Moreover, in this work, five sites were identified to be amenable to gene integrations without impacting cell growth. This finding allowed researchers to rapidly engineer a semi-synthetic lycopene biosynthetic pathway by integrating four different genes at different loci without the need for marker recovery. The integration sites could be expanded to repetitive genomic sequences from rDNA and zeta site loci, thus enabling not only the integration of multiple genes but also multi-copy integrations in these loci.

In addition, a similar method was developed in *L. starkeyi* for targeted rDNA integration of multiple copies of a hygromycin resistance gene (Oguro et al., 2015). It was found that one to five copies were integrated in different transformants, and the level of hygromycin B resistance was approximately proportional to the copy number of the integrated resistance gene. In the future, the method is expected to be used to express more target genes in *L. starkeyi*.

### Improving the Efficiency of Genetic Modifications

Despite the numerous advantages and applications of oleaginous yeasts as microbial cell factories, it is much more difficult to perform genetic modifications in oleaginous yeasts than in the model yeast *S. cerevisiae*. The difficulty is presumably due to the strong preference for non-homologous end-joining (NHEJ) and low activity for HR in oleaginous yeasts. The high capacity to undergo HR is the basis of many genetic tools and manipulations. Fortunately, it was shown that removal of the *KU70* homologs in *R. toruloides* resulted in a strain that showed an increased HR efficiency and dramatically improved gene-targeting frequency (Koh et al., 2014). Successful examples were also made in *Y. lipolytica* strains defective in NHEJ (Kretzschmar et al., 2013). Recently, a series of *L. starkeyi* mutants were constructed by disrupting genes encoding LsKu70p, LsKu80p, and/or LsLig4p, which shared sequence homology with Ku70p, Ku80p, and Lig4p, respectively, that are involved in the NHEJ pathway in other yeasts (Oguro et al., 2017). However, only the HR efficiency of the *L. starkeyi* Δ*lslig4* strain was markedly enhanced.

### Genome Editing Tools

In recent years, the type II CRISPR/Cas9 system has been widely used in biotechnology for precision genome engineering in many organisms owing to its simplicity and high efficiency (DiCarlo et al., 2013; Cobb et al., 2014; Jakočiūnas et al., 2016; Li Y. et al., 2016; Pohl et al., 2016; Wendt et al., 2016; Weninger et al., 2016). Moreover, the use of nuclease-deficient Cas9 (dCas9) enabled tunable and orthogonal control of gene expression by blocking transcription elongation (Jensen et al., 2017; Wang M. et al., 2017). More recently, CRISPR/Cas9 genome editing was developed for use in *Y. lipolytica* (Schwartz et al., 2015). Co-transformation of the CRISPR/Cas9 system and a HR donor plasmid resulted in markerless HR efficiency of over 64%. The efficiency could be increased up to 100% when NHEJ was disrupted. Later, simultaneous double and triple multigene editing by CRISPR/Cas9 was also achieved in *Y. lipolytica* (Gao S. et al., 2016). The system enabled efficient, scarless, single or multigene editing through NHEJ and HR, and should greatly facilitate future metabolic engineering of *Y. lipolytica*.

### Metabolic Models and Omics Analysis

Genome scale metabolic models (GEMs) are powerful tools to bridge the gap between genotype and phenotype. GEMs have been successfully applied to guide and design metabolic engineering strategies in many hosts (Chen et al., 2017). In 2012, two genome-scale metabolic models of *Y. lipolytica* were developed and it was shown that the predictions from these two models were consistent with published experimental data (Loira et al., 2012; Pan and Qiang, 2012). However, neither of them was used to design new metabolic engineering approaches. Recently, a new GEM of *Y. lipolytica* was reconstructed and used to optimize cell growth and lipid production (Kavšček et al., 2015). The prediction was confirmed experimentally, which yielded an 80% increase of biomass and fourfold increase of lipid yield. Later, another GEM of *Y. lipolytica* was reconstructed and used for integrative analysis of multilevel omics data, which showed that lipid accumulation in *Y. lipolytica* was associated with regulation of amino-acid biosynthesis (Kerkhoven et al., 2016). These results may enable the coupling of cell growth and lipid accumulation, which is required for obtaining high lipid content.

Kinetic models have been also developed to describe and simulate the relationship between cell growth and lipid production (Papanikolaou and Aggelis, 2003; Shen et al., 2013). A recent numerical model was built to describe the behavior and of *Cryptococcus curvatus* for improving cell and lipid production (Béligon et al., 2016). The model was used to search for optimal dilution rate and C/N ratios for continuous culture. A continuous culture was then launched using these culture parameters, resulting high cell mass and lipid productivities with respective values at 1.07 and 0.54 g/L/h. These values fitted the model predictions and were superior to previous reports for continuous cultures. Additionally, three dynamic metabolic models of *Y. lipolytica* were presented to describe its lipid accumulation and citric acid production (Robles-Rodriguez et al., 2017). Results showed a good fit of parameters on describing the dynamics of lipids and citric acid production, which proved that they can be incorporated into control strategies to optimize lipid accumulation.

Despite the above-mentioned successes, the power of modeling is still limited. Moreover, lipid metabolism is quite complex. It was found to be useful to use omics technologies to elucidate complex phenotypes (Nielsen, 2009). To get a more complete and precise picture, omics technologies will allow metabolic engineers to better understand lipid metabolism on a system level. A few studies have used omics analysis to discover and understand the metabolic regulators of lipid synthesis and accumulation in *Y. lipolytica*, such as the protein kinase Snf1 (Seip et al., 2013), a regulator of desaturase Mga2 (Liu et al., 2015b), a global regulator Mig1 (Wang et al., 2013), and the yeast TOR complexes (TORC1) (Kerkhoven et al., 2017). In addition, global responses were characterized at system-wide levels to determine how the nitrogen source regulates lipid metabolism (Zhu et al., 2012, 2015; Liu Z. et al., 2013; Kourist et al., 2015; Pomraning et al., 2016, 2017; Zhang H. et al., 2016); comprehensive changes were monitored during a transition from biomass production to lipid accumulation (Morin et al., 2011; Pomraning et al., 2015); comparative proteomics analysis was introduced to compare the non-oleaginous yeast, *S. cerevisiae*, with two oleaginous yeast strains (Shi et al., 2013). Such changes and findings were highly relevant to lipid accumulation. These omics information should aid understanding of the regulation of lipid metabolism, which will allow further metabolic engineering of oleaginous yeasts for the production of lipid-derived products.

## Engineering Oleaginous Yeasts for Production of Fuels and Chemicals

Metabolic engineering of oleaginous yeasts provides a renewable route to produce desired fuels or chemicals (**Table 1**). Some of these microorganisms may possess part of the metabolic pathway, but few contain the complete pathway or can synthesize the desired compound efficiently. Therefore, construction and optimization of the target pathways are often required.

**Table 1:** Examples of engineering oleaginous yeasts for producing a variety of different products in the recent 3 years (2015–2017).

Products		Organism	Titer	Reference
**Fatty acid-derived products**		
FAEEs	Mixture of ethyl esters of palmitic acid, palmitoleic acid, stearic acid, oleic acid, linoleic acid, arachidic acid	*Y. lipolytica*	142.5 mg/L	Xu et al. (2016)
Alkanes	Mixture of 8-heptadecene, heptadecane, 7-pentadecene, pentadecane, tridecane	*Y. lipolytica*	23.3 mg/L	Xu et al. (2016)
FALs	Hexadecanol	*Y. lipolytica*	690.21 mg/L	Wang et al. (2016a)
	Hexadecanol and octadecanol	*Y. lipolytica*	167 mg/L	Wang et al. (2016b)
	Hexadecanol and octadecanol	*L. starkeyi*	770 mg/L	Wang et al. (2016b)
	Stearic alcohol, palmitic alcohol, and oleic alcohol	*Y. lipolytica*	2.15 g/L	Xu et al. (2016)
	Decanol	*Y. lipolytica*	Over 500 mg/L	Rutter and Rao (2016)
	Oleyl alcohol, stearyl alcohol, and cetyl alcohol	*R. toruloides*	Over 8 g/L	Fillet et al. (2015)
FFAs	Decanoic and octanoic acids	*Y. lipolytica*	0.3–0.6 g/L	Rutter et al. (2015)
	Mixture of lauric acid, myristic acid, palmitic acid, palmitoleic acid, stearic acid, oleic acid, linoleic acid	*Y. lipolytica*	9.67 g/L	Xu et al. (2016)
	Myristic acid	*Y. lipolytica*	11.6% of total FAs	Rigouin et al. (2017)
	Mixture of palmitic acid, palmitoleic acid, stearic acid, oleic acid, linoleic acid, arachidic acid, behenic acid, lignoceric acid	*Y. lipolytica*	10.4 g/L	Ledesma-Amaro et al. (2016a)
PUFAs	Arachidonic acid	*Y. lipolytica*	0.4% of total FAs	Liu et al. (2017)
	γ-Linolenic acid	*Y. lipolytica*	71.6 mg/L	Sun et al. (2017)
	Conjugated linoleic acid	*Y. lipolytica*	302 mg/L	Imatoukene et al. (2017)
	Eicosatrienoic acid	*T. oleaginosus*	16% of total FAs	Görner et al. (2016)
	Eicosadienoic acid	*T. oleaginosus*	9% of total FAs	Görner et al. (2016)
	Conjugated linoleic acid	*T. oleaginosus*	2.6% of total FAs	Görner et al. (2016)
	Linoleic acid	*R. toruloides*	1.3 g/L	Wang et al. (2016d)
	α-Linolenic acid	*L. starkeyi*	126.72 mg/L	Salunke et al. (2015)
	ω-3 Eicosapentaenoic acid	*L. starkeyi*	74.28 mg/L	Salunke et al. (2015)
	Docosahexaenoic acid	*L. starkeyi*	1080 mg/L	Salunke et al. (2015)
TAGs		*Y. lipolytica*	55 g/L	Qiao et al. (2015)
		*Y. lipolytica*	66.4 g/L	Xu et al. (2016)
		*R. toruloides*	16.4 g/L from glucose; 9.5 g/l from xylose	Zhang et al. (2015)
		*R. toruloides*	89.4 g/L	Zhang S. et al. (2016)
		*Y. lipolytica*	85 g/L	Friedlander et al. (2016)
		*Y. lipolytica*	66.8 % of CDW	Qiao et al. (2017)
**Non-fatty acid-derived products**		
PHB		*Y. lipolytica*	7.35 g/L	Li Z.J. et al. (2016)
Organic acids	Citric acid	*Y. lipolytica*	111.1 g/L	Fu et al. (2016)
	Citric acid	*Y. lipolytica*	101.0 g/L	Tan et al. (2016)
	Citric acid	*Y. lipolytica*	93 g/L	Mirończuk et al. (2016)
	α-Ketoglutaric acid	*Y. lipolytica*	46.7 g/L	Guo H. et al. (2015)
	α-Ketoglutaric acid	*Y. lipolytica*	50 g/L	Guo et al. (2016)
	Succinic acid	*Y. lipolytica*	50.2 g/L	Yuzbashev et al. (2016)
	Succinic acid	*Y. lipolytica*	160 g/L	Gao C. et al. (2016)
	Succinic acid	*Y. lipolytica*	110.7 g/L	Cui et al. (2017)
	Itaconic acid	*Y. lipolytica*	4.6 g/L	Blazeck et al. (2015)
Erythritol		*Y. lipolytica*	78 g/L	Mirończuk et al. (2016)
Erythritol		*Y. lipolytica*	80.6 g/L	Carly et al. (2017)
Terpenoids	α-Farnesene	*Y. lipolytica*	259.98 mg/L	Yang et al. (2016)
	Limonene	*Y. lipolytica*	23.56 mg/L	Cao et al. (2016)
	Campesterol	*Y. lipolytica*	453 mg/L	Du et al. (2016)
	Campesterol	*Y. lipolytica*	942 mg/L	Zhang et al. (2017)
	β-Carotene	*Y. lipolytica*	4 g/L	Gao et al. (2017)
	Carotenoids	*R. toruloides*	2.9 μg/mg CDW	Lee et al. (2016)

### Production of FA-Derived Products

**FIGURE 1:**
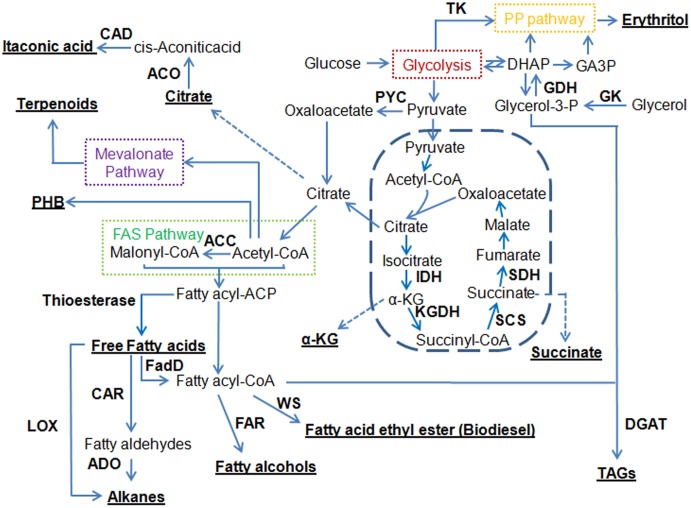
Overview of metabolic pathways for engineering oleaginous yeasts to produce a variety of different compounds. Underlined are target compounds produced by oleaginous yeasts. ACC, acetyl-CoA carboxylase; ACO, aconitase; ADO, aldehyde deformylating oxygenase; CAD, *cis*-aconitic acid decarboxylase; CAR, carboxylic acid reductase; DGAT, diacylglycerol acyltransferase; DHAP, dihydroxyacetone phosphate; FadD, fatty acyl-CoA synthetase; FAR, fatty acyl-CoA reductase; FAS pathway, fatty acid synthase pathway; GA3P, glyceraldehyde-3-phosphate; GDH, glycerol-3P dehydrogenase; GK, glycerol kinase; IDH, isocitrate dehydrogenase; α-KG, α-ketoglutarate; KGDH, ketoglutarate dehydrogenase; LOX, lipoxygenase; PHB, poly-3-hydroxybutyrate; PP pathway, pentose phosphate pathway; PYC, pyruvate carboxylase; SCS, succinyl-CoA synthase; SDH, succinate dehydrogenase; TAGs, triacylglycerols; TK, transketolase; WS, wax ester synthase.

FAs are natural precursors to many classes of compounds. **Figure 1** shows that a number of fuels and chemicals can be derived from FAs or their biosynthetic intermediates by introducing the corresponding conversion steps.

FAEEs can be used as biodiesel, which is considered as one of the most prominent renewable energy resources. As shown in **Figure 1**, the microbial *de novo* biosynthesis of FAEEs was achieved by expressing a wax ester synthase (WS) (Steen et al., 2010; Shi et al., 2012). Recently, Xu et al. (2016) demonstrated that expression of *Acinetobacter baylyi* ADP1 WS in endoplasmic reticulum led to an engineered *Y. lipolytica* strain producing 142.5 mg/L FAEEs. Besides FAEEs, alkanes, a more ideal substitute for fossil diesels, were produced using metabolically engineered strains (Schirmer et al., 2010). Blazeck et al. (2013) reported and characterized a proof-of-concept pathway that enabled production of the C5 *n*-alkane at 4.98 mg/L in *Y. lipolytica* by utilizing a soybean lipoxygenase enzyme. Recently, up to 23.3 mg/L of alkanes were made by expressing the carboxylic acid reductase from *Mycobacterium marinum* and the aldehyde deformylating oxygenase from *Prochlorococcus marinus* (Xu et al., 2016). These studies suggested that the endogenous FA pool may be an alternative route to synthesizing FAEEs or alkanes. However, the titers of FAEEs and alkanes produced are low, suggesting that future efforts focusing on further strain improvement, bioprocess optimization, and enzyme engineering are still needed.

FALs represent a range of aliphatic alcohols with chain lengths ranging from C8 to C32, and FALs can be used in the formulation of various types of products, including fuels, lubricants, surfactants, solvents, cosmetics, personal care products, pharmaceuticals, and plastics. FALs can be derived by the reduction of different acyl-CoA molecules to the corresponding primary alcohols (**Figure 1**). The capability of producing FALs in oleaginous yeasts has not been explored until recently. Specifically, Wang et al. (2016a) introduced a functional fatty acyl-CoA reductase from *Tyto alba* (TaFAR1) to direct the conversion from fatty acyl-CoA to FALs in *Y. lipolytica*. Up to 690.21 mg/L hexadecanol was produced by this cell factory through batch fermentation. In parallel, FALs can also be directly produced by expression of the FAR gene from *Marinobacter aquaeolei* VT8 (Wang et al., 2016b). This strategy resulted in the production of 167 and 770 mg/L of FALs (mainly hexadecanol and octadecanol) in shake flask from *Y. lipolytica* and *L. starkeyi*, respectively. Currently, in *Y. lipolytica*, the most efficient synthesis of FALs was achieved by activation of endogenous FFAs and the subsequent reduction of fatty acyl-CoAs (Xu et al., 2016). In particular, expression of the *M. aquaeolei* FAR along with an *E. coli* fatty acyl-CoA synthetase (EcfadD) led to dramatic titer improvement of FALs to 2.15 g/L in a 3-L bioreactor. It was also found that the chain length of FALs can be controlled by introduction of thioesterases and an FAR in *Y. lipolytica*, enabling the production of medium-chain FALs with titers exceeding 500 mg/L (Rutter and Rao, 2016). *R. toruloides* is an important oleaginous yeast with a significantly higher lipid content compared to *Y. lipolytica*. It was reported that over 8 g/L of C16–C18 FALs were produced in *R. toruloides* by expressing a FAR from *M. aquaeolei* VT8 (Fillet et al., 2015). This is the highest titer ever reported on microbial production of FALs to date, and it only needs one genetic manipulation, demonstrating that this oleaginous yeast is a promising host to produce long-chain FALs and other oleochemicals.

Molecules of FFAs of different structures appear to be excellent precursors for the application in the production of custom biofuels or chemicals (**Figure 1**). It has been shown that the profile of FFAs can be efficiently modified in the chain length and the degree of unsaturation (Lennen and Pfleger, 2012). Medium chain-length FAs could be produced by expressing five codon-optimized plant and bacterial fatty acyl-ACP thioesterases in *Y. lipolytica* (Rutter et al., 2015), which in turn produced medium-chain FALs (Rutter and Rao, 2016). Xu et al. (2016) fused the truncated FAS1 with putative thioesterases in *Y. lipolytica*, which resulted in an outstanding titer of FFAs at 9.67 g/L. The resulting strain had a remarkably increased C12 and C14 portions of FAs, accounting for 7.5 and 29.2% of total FAs, respectively. Beyond the use of thioesterases, a FA synthase (FAS) was engineered to shorten the chain length of the synthesized FAs, which led to an accumulation of myristic (C14) acid at a level of 11.6% of total FAs (Rigouin et al., 2017). Secretion of FFAs into the medium could help avoid toxicity and save on extraction costs. For the first time in oleaginous organisms, in particular in *Y. lipolytica*, Ledesma-Amaro et al. (2016a) released FFAs from the lipid bodies by overexpressing different intracellular lipases, and developed a strain with a FFA production at 10.4 g/L. These studies have established the basis for future genetic manipulations to boost the production and reduce the cost for lipid extraction through the secretion of FFAs.

In oleaginous yeasts, most of the lipids are accumulated in the form of TAGs. TAGs may serve as a renewable source of oil and are well-suited as an intermediate building block for fuels and chemicals. There are numerous reports in the engineering of *Y. lipolytica* for increasing the yield of TAGs and these attempts have been mainly focused on the biosynthetic pathways of TAGs (Dulermo and Nicaud, 2011; Tai and Stephanopoulos, 2013; Blazeck et al., 2014). Similarly, overexpression of native acetyl-CoA carboxylase (ACC) and diacylglycerol acyltransferase genes also increased lipid production in *R. toruloides* (Zhang et al., 2015). The engineered strain was able to produce 16.4 g/L lipid from glucose and 9.5 g/L lipid from xylose. Later, the same group managed to further increase its lipid production to 89.4 g/L through the overexpression of stearoyl-CoA desaturase (SCD; Zhang S. et al., 2016). Recently, analysis of gene expression in specialized mammalian lipid-storing tissues identified the Δ-9 SCD as a rate limiting step for the metabolic engineering of the TAG synthesis pathway (Qiao et al., 2015). Simultaneous expression of the *SCD*, *ACC*, and *DGA1* genes led to an engineered *Y. lipolytica* strain with high lipid titer (55 g/L). Moreover, the engineered strain also exhibited several favorable phenotypes including fast growth and high sugar tolerance. To take it a step further, this group engineered five alternative cytosolic acetyl-CoA pathways in *Y. lipolytica* (Xu et al., 2016). The best performer was the strain carrying the acetyl-CoA shuttling pathway (carnitine acetyltransferase Cat2), which achieved a dry cell weight of 91.6 g/L and a lipid titer of 66.4 g/L. To identify more genes that contributed to the improvement of lipid production, the same group evaluated the effect of the overexpression of a set of 44 native genes on lipid production in *Y. lipolytica* (Silverman et al., 2016). By overexpressing a single gene at one time, a set of genes were isolated that were effective at individually influencing lipid production. These included the *DGA2* and *SLC1* genes that directly catalyzed the reactions of lipid synthesis, the *GPD1* gene that increased production of glycerol head groups and the *SOL3* gene that increased NADPH availability. In another attempt to increase lipid accumulation in *Y. lipolytica*, Friedlander et al. (2016) optimized key enzymes by screening heterologous genes to create an improved lipid-accumulating biocatalyst. The identified genes, *DGA1* from *R. toruloides* and *DGA2* from *Claviceps purpurea*, were co-expressed in a strain lacking TLG3 activity, an intracellular lipase responsible for the degradation of TAGs, which yielded an impressive lipid titer of 85 g/L. A mathematical model was established and identified the extent to which the yield of lipid production can be obtained in *Y. lipolytica* (Qiao et al., 2017). Stearic acid (SA) was chosen as the end product to simplify the model. If it is assumed that excess reducing equivalents generated in the form of NADH can be converted to the cytosolic NADPH, a maximum yield can be calculated as 0.344 g-SA/g-glucose in comparison to 0.271 g/g glucose in native *Y. lipolytica*. Accordingly, four synthetic pathways were designed to convert NADH to NADPH. The best strain showed a lipid content at 66.8% and exhibited the titer and productivity of FAMEs to 99 g/L and 1.2 g/L/h, respectively. The high yields, productivities, and titers reported in these studies suggest that it is feasible to develop cost effective, large-scale microbial lipid production processes.

**FIGURE 2:**
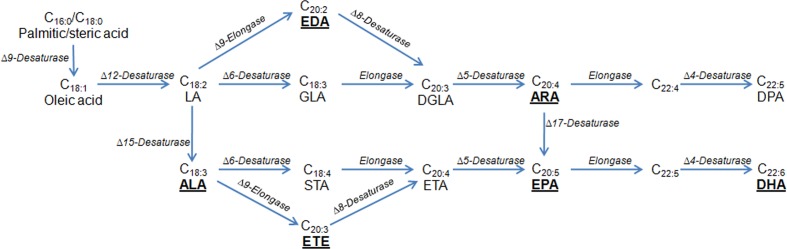
Biosynthetic pathways for the production of PUFAs. Underlined are PUFAs produced by oleaginous yeasts. The pathway can be classified into a Δ6-desaturase pathway or a Δ9-elongase and Δ8-desaturase pathways (the Δ9 pathway). In the Δ6 pathway, the first step is the Δ6 desaturase to covert the LA and/or ALA to GLA and/or STA; the second step is the C18/20 elongase to convert the GLA and/or STA to DGLA and/or ETA. In the Δ9 pathway, the first step is the Δ9 elongase to convert LA and/or ALA to EDA and/or ETE; the second step is the Δ8 desaturase to convert EDA and/or ETE to DGLA and/or ETA. The last two steps are the same between these two pathways. ALA, α-linolenic acid; ARA, arachidonic acid; DGLA, dihomo-γ-linolenic acid; DHA, docosahexaenoic acid; DPA, docosapentaenoic acid; EDA, eicosadienoic acid; EPA, ω-3 eicosapentaenoic acid; ETA, eicosatetraenoic acid; ETE, eicosatrienoic acid; GLA, γ-linolenic acid; STA, stearidonic acid; LA, linoleic acid.

In addition, many researchers have successfully produced specialty oils containing PUFAs used in the food and supplement industries. PUFA biosynthesis is generally associated with a variety of pathways of desaturation and elongation (**Figure 2**), and all the genes involved in PUFA biosynthesis have been identified from multiple organisms. This has made it possible to accumulate tailored PUFAs in heterologous hosts. As a first example of successful commercialization, *Y. lipolytica* has been used industrially to produce ω-3 eicosapentaenoic acid (EPA), making a breakthrough to replace an animal-derived product (Xie et al., 2015). Recently, ARA, a typical omega-6 PUFA, was synthesized via the aerobic Δ-6 desaturation and elongation pathway in *Y. lipolytica* (Liu et al., 2017). In the engineered strain, a high level of ARA production (0.4% of total FAs) was achieved. Similarly, the same group also engineered *Y. lipolytica* for the production of γ-linolenic acid (GLA) (Sun et al., 2017). An optimized GLA production at 71.6 mg/L was obtained by applying a novel temperature-shift strategy. In another study, up to 302 mg/L of conjugated linoleic acid was produced in the engineered strain of *Y. lipolytica* via various genetic modifications (Imatoukene et al., 2017), including elimination of β-oxidation, removal of the ability to store lipids as triglycerides, and the overexpression of the Δ12-desaturase gene. At the same time, Görner et al. (2016) evaluated the ability of *T. oleaginosus* to generate non-natural FA profiles by heterologous expression of several FA modifying enzymes. Yeast strains were designed to produce the polyunsaturated very long chain FAs eicosatrienoic at 16% of total FAs and eicosadienoic acid at 9% of total FAs, respectively. In this study, *T. oleaginosus* was also engineered to produce the non-native conjugated linoleic acid (2.6% of total FAs). In addition, transformed *L. starkeyi* with flax Δ15 desaturase enabled conversion of linoleic acid into α-linolenic acid (ALA) at 126.72 mg/L, and the ALA produced was utilized further in this yeast leading to accumulation of EPA (74.28 mg/L) and docosahexaenoic acid (1080 mg/L) (Salunke et al., 2015). In *R. toruloides*, the relative linoleic acid content was increased up to fivefold and the final linoleic acid titer reached 1.3 g/L under flask culture conditions by galactose-inducible expression of the gene encoding Δ12-desaturase from *Mortierella alpina* or *Fusarium verticillioides* (Wang et al., 2016d). These works demonstrated that oleaginous yeasts presented novel opportunities for the production of designed and high value FAs.

### Production of Acetyl-CoA Derived, Non-FA Products

The process of lipid accumulation starts from acetyl-CoA, and a high lipid content requires an efficient supply of acetyl-CoA. This is the basis for investigating oleaginous yeasts as a preferred platform for production of acetyl-CoA derivative products.

Terpenoids are a large family of natural products and many of them have been widely applied in the pharmaceutical and nutritional industries, such as paclitaxel and artemisinin. Terpenoids can also be used as biofuels due to their branched hydrocarbon chains and various ring structures. As shown in **Figure 1**, the biosynthetic pathways of terpenoids start from acetyl-CoA via the mevalonate pathway. Terpenoids have been the target of metabolic engineering in *E. coli* or *S. cerevisiae* (Farhi et al., 2011; Wang C. et al., 2017), and researchers have also started to explore the synthesis of terpenoids in oleaginous yeasts. Recently, α-farnesene, a potential bio-jet fuel molecule, was produced in an engineered *Y. lipolytica* by overexpressing a codon-optimized apple α-farnesene synthase gene and genes in the mevalonate pathway (Yang et al., 2016). The engineered strain produced 259.98 mg/L of α-farnesene with a yield at 33.98 mg/g, which was the highest ever reported in yeast. Around the same time, the limonene biosynthesis was reported in an engineered *Y. lipolytica* for the first time (Cao et al., 2016). In the engineered strain, two genes encoding neryl diphosphate synthase 1 and limonene synthase were codon-optimized and heterologously expressed together with the overexpression of several genes involved in the mevalonate pathway. The produced limonene reached 23.56 mg/L, which was the highest level reported in yeast. Moreover, campesterol biosynthesis was also realized in *Y. lipolytica* (Du et al., 2016). The engineered strain disrupted ergosterol formation and constitutively expressed the heterologous 7-dehydrocholesterol reductase (DHCR7), and achieved a titer of 453 mg/L, which was much higher than what was reported in *S. cerevisiae* (Souza et al., 2011). The authors found that the enzyme DHCR7 played an important role in enhancing the production of campesterol. Thus, more DHCR7 enzymes from diverse species were investigated, and the DHCR7 from *Danio rerio* was the best candidate for campesterol synthesis (Zhang et al., 2017). Together with an overexpression of POX2 (peroxisome acyl-CoA oxidase 2), the production of campesterol finally reached 942 mg/L. Meanwhile, the β-carotene and its precursor lycopene were also found to accumulate in the lipid bodies of engineered *Y. lipolytica* (Matthäus et al., 2014; Gao et al., 2017). Specifically, the production of β-carotene at 4 g/L was the highest titer reported to date, and it was achieved by overexpression of its biosynthetic pathway using strong promoters and multiple gene copies for each of the 12 steps (Gao et al., 2017). It is widely known that the oleaginous yeast *R. toruloides* can naturally accumulate high levels of carotenoids. Recently, a membrane transporter Pdr10 was introduced into *R. toruloides* to facilitate production and separation of carotenoids (Lee et al., 2016). In the resulting strain, a total of 2.9 μg/mg carotenoids was produced, while a total of 1.8 μg/mg carotenoids was exported. This strategy eliminates the need for product extraction and may be applied to other organisms producing terpenoids or other lipids.

The production of PHB is highly dependent on the intracellular availability of acetyl-CoA and reducing equivalent NADPH. Recently, the PHB biosynthetic pathway was expressed in *Y. lipolytica* (Li Z.J. et al., 2016). In pH controlled acetate fed-batch fermentation, 7.35 g/L PHB was produced, which was the highest PHB production reported in yeast. The study demonstrated the fact that a good lipid producer could guarantee the supply of acetyl-CoA and in turn facilitate the production of its derivatives.

### Production of Other Unique Products

Oleaginous yeasts have also emerged as a preferred platform for production of other products due to their unique features. For example, *Y. lipolytica* was used to produce organic acids due to its innate ability to accumulate citric acid (Akiyama et al., 1973) and its tolerance to low pH (Liu et al., 2015a). There were many reports on citric acid production in *Y. lipolytica* using various substrates (Liu et al., 2015a). Recently, a pyruvate carboxylase (*PYC1*) from *Penicillium rubens* I607 was expressed in *Y. lipolytica* to catalyze an increase in the formation of oxaloacetic acid, which in turn led to the production of more citric acid (Fu et al., 2016). The corresponding recombinant *Y. lipolytica* strain was able to produce citric acid at 111.1 g/L within 240 h, which was higher than that produced by most of other engineered yeast strains. Similarly, the same group also expressed another PYC, derived from *Meyerozyma guilliermondii*, in *Y. lipolytica* for citric acid production, reaching 101.0 g/L (Tan et al., 2016). These two studies also demonstrated the key role of PYC in the citric acid production.

α-Ketoglutarate (α-KG) can be synthesized by isocitrate dehydrogenase in the TCA cycle, and ketoglutarate dehydrogenase (KGDH) complex catalyzes the oxidation of α-KG to succinyl-CoA (**Figure 1**). The microbial production of α-KG was previously described by a review (Otto et al., 2011). Recently, six putative transporter genes were evaluated in *Y. lipolytica* to assess their roles in regulating extracellular keto acids accumulation (Guo H. et al., 2015). In the strain containing the transporter YALI0B19470g, there was a significant increase in α-KG production (up to 46.7 g/L) with a sharp decrease in by-product accumulation, suggesting a new and promising strategy that can efficiently address accumulation of organic acids. Later, the same group weakened the activity of KGDH to reduce the consumption of α-KG by mutating the inner core of KGDH (Guo et al., 2016). This strategy led to a 40% increase of α-ketoglutarate production (50 g/L). As the KGDH complex plays a critical role in the central carbon metabolism, their observations could provide a general strategy for regulating the carbon flux.

Succinic acid is another important organic acid with applications in food, chemical, and agricultural industries. A *Y. lipolytica* strain with a defective succinate dehydrogenase (SDH) was constructed, which produced 17.5 g/L of succinic acid (Yuzbashev et al., 2010). The subsequent directed evolution experiment generated a mutant strain capable of producing succinic acid at 50.2 g/L (Yuzbashev et al., 2016). Similarly, Gao C. et al. (2016) disrupted SDH to construct a succinate-production strain. After 400 h cultivation, the strain achieved a succinic acid production at 160 g/L, which was the highest titer obtained in fermentation on succinic acid production. However, the authors noticed that the strain also produced a large amount of acetate during the fermentation process, affecting the cell growth and succinate production yield. In their follow-up study, the strain was further engineered by eliminating acetic acid formation and overexpressing the genes that can improve the formation of succinic acid through reductive carboxylation (Cui et al., 2017). Finally, a succinic acid titer of 110.7 g/L was achieved in 138 h with a significant reduction in the formation of acetic acid.

Itaconic acid is another promising organic acid with diverse applications, including as a replacement for petroleum-derived products. *Y. lipolytica* is viewed as an alternative host for itaconic acid production due to its proven potential in the efficient supply of citric acid (Wang et al., 2012), which can be converted to itaconic acid in two steps by aconitase (ACO) and *cis*-aconitic acid decarboxylase (CAD) (**Figure 1**). Recently, it was shown that a high level of itaconic acid (4.6 g/L) could be produced in *Y. lipolytica* by overexpression and cytosolic co-localization of CAD and ACO (Blazeck et al., 2015), suggesting that *cis*-aconitic acid permeability through the mitochondrial membrane was limiting compared to citric acid. Furthermore, this titer was achieved by using an inexpensive, minimal media that could be beneficial for downstream processing.

Erythritol is a biological sweetener with wide applications in food and pharmaceutical industries, which can be produced via chemical or biological approaches (Moon et al., 2010). *Y. lipolytica* represents a promising host for erythritol production, especially using glycerol as the carbon source (**Figure 1**). For example, Mirończuk et al. (2016) engineered *Y. lipolytica* for erythritol production from glycerol by overexpression of glycerol kinase (GK) and glycerol-3-P dehydrogenase (GDH). In this work, the production of erythritol achieved 78 g/L with a productivity of 1.08 g/L/h. Similarly, a pull and push metabolic engineering strategy was used to improve the erythritol production (Carly et al., 2017). The best results were obtained by overexpression of GK and transketolase, and in which EYK1, which is involved in an early step of erythritol catabolism, was disrupted. In the engineered strain, the titer of erythritol reached 80.6 g/L while its productivity reached 1.03 g/L/h.

### Utilization of Low-Cost Substrates

Feedstock accounts for the majority of the production cost in the fermentation processes. Since many strains cannot efficiently grow in most of the readily available inexpensive carbon sources, to realize an economically viable microbial production process, more efforts are required to engineer oleaginous yeasts to efficiently utilize renewable and inexpensive carbon sources. One of such carbon sources is plant lignocellulosic biomass which is the most abundant renewable resource on earth. Microbial utilization of lignocellulosic biomass is viewed as a crucial part of the bioeconomy (den Haan et al., 2015). Lignocellulose is mainly composed of cellulose, hemicellulose, and lignin.

**FIGURE 3:**
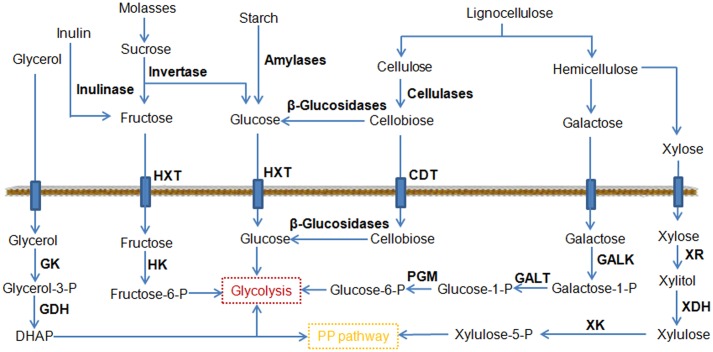
Schematic representation of the pathways leading to the consumption of an expanded range of substrates by metabolic engineering. CDT, cellodextrin transporter; GALK, galactokinase; GALT, galactose-1-phosphate uridylyltransferase; GDH, glycerol-3P dehydrogenase; GK, glycerol kinase; HK, hexokinase; HXT, hexose transporter; PGM, phosphoglucomutase; XDH, xylitol dehydrogenase; XK, xylulokinase; XR, xylose reductase.

Cellobiose is a glucose dimer obtained from cellulose (**Figure 3**), and its utilization is a rate-limiting step in the consumption of cellulose. Cellobiose utilization in *Y. lipolytica* has been demonstrated by chromosomal expression of cellodextrin transporter (*cdt-1*) and intracellular β-glucosidase (*gh1-1*) (Lane et al., 2015). In another study, six versions of β-glucosidases were investigated for their ability to use cellulose (Guo Z. et al., 2015). Two strains overexpressing *BGL1* and *BGL2* encoding β-glucosidase were able to degrade cellobiose. Significantly, the strain co-overexpressing *BGL1* and *BGL2* grew better than the *Y. lipolytica* strains expressing single *BGLs*. By further expression of a cellulase cocktail, the resulting engineered *Y. lipolytica* strain was able to grow both on model cellulose substrates, such as highly crystalline Avicel, and on industrial cellulose pulp, such as that obtained using an organosolv process (Guo et al., 2017). The good performance of the strain on an industrial cellulose substrate revealed that this yeast strain could be a vital step toward the development of a next-generation biorefinery process.

Hemicelluloses are heterogeneous polymers consisting of a mixture of pentoses (xylose and arabinose) and hexoses (mannose, glucose, and galactose). Among them, xylose is the second most abundant sugar in lignocellulosic hydrolysates after glucose (Jeffries and Jin, 2004). Many industrial microorganisms cannot naturally metabolize xylose. A great deal of effort has been directed toward engineering microorganisms for xylose utilization, which led to robust xylose utilization in *Y. lipolytica* (Ledesma-Amaro and Nicaud, 2016b). However, most of these engineered strains did not grow well on xylose. Recently, it is found that the endogenous xylulokinase (XK) gene limits *Y. lipolytica*’s growth on xylose (Ledesma-Amaro et al., 2016b). The overexpression of *Scheffersomyces stipitis* xylitol dehydrogenase (XDH) and xylose reductase (XR) was necessary but not sufficient to permit growth. The additional overexpression of the endogenous XK enabled identical growth as the wild type strain in glucose, achieving a citric acid titer of 80 g/L. At the same time, *Y. lipolytica* was engineered to utilize xylose as a sole carbon source and produce over 15 g/L of lipid by firstly introducing the heterologous *XR* and *XDH* genes and then enabling gene duplication through starvation (Li and Alper, 2016). Confirmed by genome sequencing, it was found that this efficient phenotype was predominately enabled by gene duplications to allow for higher expression of XR and XDH. As mentioned before, galactose is another monosaccharide found in hemicelluloses. The Leloir metabolic pathway can convert galactose to glucose-6-phosphate, which enters glycolysis (**Figure 3**). Recently, a *Y. lipolytica* strain was created to efficiently utilize galactose as its sole carbon source by fully activating the Leloir pathway (Lazar et al., 2015). Notably, the citric acid and lipid production by this modified yeast grown in galactose was similar to or greater than that when grown in glucose, making it possible to efficiently exploit lignocellulosic biomass for biotechnological applications.

Molasses is one of the cheapest carbon feedstocks currently available for industrial fermentation, and is composed predominantly of sucrose (**Figure 3**). The expression of invertase allowed a rapid cleavage of sucrose into glucose and fructose in *Y. lipolytica* (Lazar et al., 2013). To use molasses as a substrate, the *S. cerevisiae SUC2* gene (encoding invertase) was expressed in *Y. lipolytica* (Gajdoš et al., 2015). The engineered strain reached a final biomass yield at 26.6 g/L and a total FA at 8 g/L from molasses. In parallel, it was reported that fructose uptake was successfully improved by overexpressing hexokinase (Lazar et al., 2014). The same group also individually screened members of the sugar transporter family for their hexose transport ability using an appropriate heterologous host and identified two active fructose transporters in *Y. lipolytica* (Lazar et al., 2017). Based on these findings, one promising strain of *Y. lipolytica* was developed to consume different hexoses via a combination of the above-mentioned strategies (Hapeta et al., 2017). The highest values for lipid concentration and yield of lipids from the fructose reached 20.3 g/L and 0.14 g/g, respectively.

Starch is a cheap, renewable, and fermentable carbon source widely found in plants such as wheat, maize, rice, and potato. Starch consists of glucose monomers joined by glycosidic bonds (**Figure 3**). The use of starch as the substrate has already been demonstrated in *S. cerevisiae* (Aydemir et al., 2014). Recently, a *Y. lipolytica* strain was engineered to consume starch by expressing and secreting rice α-amylase and *Aspergillus niger* glucoamylase (Ledesma-Amaro et al., 2015). The strain was able to accumulate large amounts of lipids (2.29 g/L), and the lipid content was further increased to 2.84 g/L by addition of a second copy of each amylolytic enzyme. This result suggests that the ability of utilizing starch substrate might cover a wide variety of yeast species ranging from ethanol fermenting strains to oleaginous strains.

Inulin is a polymer of fructans consisting of a linear chain of fructose residues (**Figure 3**), which is widely distributed in nature as the roots or tubers of plants. Currently inulin is an interesting candidate as a renewable raw material for industrial applications. *Y. lipolytica* strains capable of metabolizing inulin were obtained by expressing the inulinase from *Kluyveromyces marxianus* (Rakicka et al., 2016). These genetically engineered strains also showed an excellent ability to produce erythritol (120.9 g/L) and citric acid (105.2 g/L). In another oleaginous yeast *Trichosporon cutaneum*, it was found that inulin could be utilized directly for microbial lipid fermentation without a hydrolysis step (Wang et al., 2015). Correspondingly, a consolidated bioprocessing technology for lipid production from inulin was developed and 4.79 g/L of lipid was produced from 50 g/L inulin.

Glycerol is a major byproduct of industrial processes such as the production of biodiesel and has been regarded as a cheap substrate for microbial production of valuable metabolites. In particular, glycerol is a very attractive substrate for lipid production because it serves as a scaffold in the formation of TAG (Gajdoš et al., 2017). Several studies were reported to improve glycerol utilization through metabolic engineering. In a recent work, Mirończuk et al. (2016) engineered *Y. lipolytica* to efficiently consume glycerol by overexpression of GK and GDH, and the modified strain resulted in a rapid biosynthesis of citric acid. The accumulated citric acid titer reached 93 g/L from glycerol. Moreover, the production of citric acid was shortened within 72 h and the productivity was 1.29 g/L/h. The short fermentation time and utilization of glycerol should be beneficial for industrial applications.

## Perspectives

Oleaginous yeasts have attracted considerable interest for their utility in the production of target compounds (**Table 1**), especially lipid-related products, from a variety of carbon sources. For instance, using *Y. lipolytica* to produce a variety of lipid-derived compounds has been investigated in an European project, called “lipoYeasts” (Sabirova et al., 2011), and using *Y. lipolytica* to produce EPA has been commercialized by DuPont (Xie et al., 2015). However, metabolic engineering of oleaginous yeasts is still in its infancy and the cost of the fermented products is still too high, which limits their commercialization. One of the main reasons is low flux toward synthesis of target compounds due to the low activity of heterologous pathways. In this regard, future efforts should be invested in the discovery or engineering of novel enzymes with higher activity, stability, and specificity. Meanwhile, homologous and heterologous pathways need to be further optimized and balanced to reach high yield and productivity. It is also important to note that improved lipid production is usually accompanied by a decrease in cell growth (Tai and Stephanopoulos, 2013; Blazeck et al., 2014). Future studies should focus on a more balanced metabolism, such as using an evolutionary approach (Liu et al., 2015c) or GEM predictions to access a middle ground for these two competing factors (Kerkhoven et al., 2016).

In the study of these oleaginous yeasts, the ability to manipulate their genes is essential for understanding their metabolism and rapid strain development. To do so, a variety of highly efficient genetic tools are needed. The development of genetic tools has been focused on promoters, terminators, standardized integration sites, pathway assembly, vectors, GEMs, and CRISPR-based systems. Although there have been a lot of achievements as shown above, especially in *Y. lipolytica*, compared to the model microorganisms such as *S. cerevisiae*, oleaginous yeasts still lag significantly in terms of genetic engineering and synthetic biology. Extension or discovery of genetic tools from model microorganisms to oleaginous yeasts would enable more rapid and convenient strain engineering and facilitate reaching the full potential of these yeasts.

## Author Contributions

SS conceived this article, reviewed the existing literature, participated in writing, and created the figures. HZ participated in conceiving this article, writing and critically reviewed all content.

## Conflict of Interest Statement

The authors declare that the research was conducted in the absence of any commercial or financial relationships that could be construed as a potential conflict of interest.
